# QSBR Study of Bitter Taste of Peptides: Application of GA-PLS in Combination with MLR, SVM, and ANN Approaches

**DOI:** 10.1155/2013/501310

**Published:** 2013-11-25

**Authors:** Somaieh Soltani, Hossein Haghaei, Ali Shayanfar, Javad Vallipour, Karim Asadpour Zeynali, Abolghasem Jouyban

**Affiliations:** ^1^Biotechnology Research Center and Faculty of Pharmacy, Tabriz University of Medical Sciences, Tabriz 51664, Iran; ^2^Hematology and Oncology Research Center, Tabriz University of Medical Sciences, Tabriz 51664, Iran; ^3^Liver and Gastrointestinal Diseases Research Center, Students' Research Committee, Tabriz University of Medical Sciences, Tabriz 51664, Iran; ^4^Tuberculosis and Lung Disease Research Center, Tabriz University of Medical Sciences, Tabriz 51664, Iran; ^5^Faculty of Chemistry, University of Tabriz, Tabriz 51664, Iran; ^6^Drug Applied Research Center and Faculty of Pharmacy, Tabriz University of Medical Sciences, Tabriz 51664, Iran

## Abstract

Detailed information about the relationships between structures and properties/activities of peptides as drugs and nutrients is useful in the development of drugs and functional foods containing peptides as active compounds. The bitterness of the peptides is an undesirable property which should be reduced during drug/nutrient production, and quantitative structure bitter taste relationship (QSBR) studies can help researchers to design less bitter peptides with higher target efficiency. Calculated structural parameters were used to develop three different QSBR models (i.e., multiple linear regression, support vector machine, and artificial neural network) to predict the bitterness of 229 peptides (containing 2–12 amino acids, obtained from the literature). The developed models were validated using internal and external validation methods, and the prediction errors were checked using mean percentage deviation and absolute average error values. All developed models predicted the activities successfully (with prediction errors less than experimental error values), whereas the prediction errors for nonlinear methods were less than those for linear methods. The selected structural descriptors successfully differentiated between bitter and nonbitter peptides.

## 1. Introduction 

Proteins are made from peptide fragments that are well known for their nutrient, biological, and physiological roles in the human body. Peptides modulate the health-connected physiological process of the cardiovascular, nervous, immune, and nutritional systems [1]. The investigation of properties and activities of peptides as therapeutic, bioactive agents and nutrients as well as starting points for the development of drugs and drug-related compounds is one of the most interesting and demanding fields of food and drug sciences. It allows researchers to compile data sets on their structures and properties/activities. The results of these studies are useful in the development of functional foods containing peptides as active compounds and drugs [2]. Peptide bitterness is an undesirable property that is frequently generated during the enzymatic process to produce functional, bioactive protein hydrolysates or during the aging process in fermented food products [3]. Since many toxins are bitter, most mammalians including humans are instinctively averse to bitter-tasting substances in order to avoid toxin ingestion [4]. Most therapeutic peptides cannot be administered orally because of the poor biopharmaceutical performance of high-molecular-weight peptide drugs which is due to poor oral absorption, formulation stability, and degradation in the gastrointestinal tract. Studies on the origins of formulations and alternative administrations to overcome the mentioned problems have suggested different administration methods such as parenteral, oral, transdermal, nasal, pulmonary, rectal, ocular, buccal, and sublingual drug delivery systems [5–8]. Taste plays a crucial role in buccal and sublingual administration systems. Bitter taste properties in relation with the structure of the peptides in fermented food and protein hydrolyzates have been studied. Findings have shown that hydrophobicity is correlated with bitterness, and a hydrophobic interaction is needed for the bitter receptors (T_2_Rs) to sense bitterness, whereas the amino acid sequence has no effect on bitterness [9, 10]. Moreover, introducing amino acids into the hydrophobic chain intensifies bitterness, and blocking both C and N terminals of peptides by acetylating increases bitterness about ten times [4]. It is now generally accepted that the side-chain hydrophobicity and the number of carbon atoms of the hydrophobic side chain of the peptide's amino acids are correlated to bitterness rather than to overall hydrophobicity [4, 9, 11, 12]. In fact, the hydrophobic group of the side chain offers a binding site for the bitter taste receptor. Another binding site is a bulky basic group, including an *α*-amino group. To provide such features, hydrophobic amino acids at the C-terminal and basic amino acids at the N-terminal are necessary. The bitter peptides are composed of less than eight amino acids, and the bitterness increases as the number of amino acids increases. Peptides composed of eight or more amino acids do not differ in bitter potency, and they form a spherical shape rather than a helix conformation [4, 9].

Beyond structure-activity studies, researchers have tried to develop quantitative models to predict the bitterness of peptides [3] and to evaluate the effect of the primary structure on the potency of therapeutic peptides [1, 13]. These studies were aimed at understanding the peptide structures responsible for different activities (taste, ACE inhibition, antithrombotic, opioid and endopeptidase inhibitory, antimicrobial activities, and immune modulator) and to accelerate the studies about food-derived functional and bioactive peptides. 

Different quantitative structure bitter taste relationship (QSBR) models using various descriptors have been developed for di- and tripeptides [1, 3, 13–16]; the study of tetra- and higher peptides is limited [3]. Some methods developed to predict the bitterness of dipeptides used amino acid-based descriptors, which resulted in models with acceptable prediction capabilities. 

Yin et al. [17] reported 28 developed models in the year 2010 for modeling dipeptide bitterness in comparison with their own model which used E_1_–E_5_ amino acid variables (hydrophobicity (E_1_), steric properties or side chain bulk/molecular size (E_2_), preferences for amino acids to occur in *α*-helices (E_3_), composition (E_4_), and the net charge (E_5_)) to develop QSBR models using support vector regression (SVM). Their model [17] successfully predicted (*R* ^2^ = 0.97) the bitterness of 48 dipeptides (*R* ^2^ values calculated using ((A.6)) of appendix).  Table 1 (28 models taken from a reference [17] + 2 other models) summarizes the developed models for dipeptides along with their prediction errors (root mean square errors (RMSE) which were calculated using ((A.1)) of the appendix).

Kim and Li-Chan (2006) [3] studied the QSBR of 224 di- to tetradecapeptides (2–14 amino acids peptides) using multiple linear regression (MLR) and partial least square (PLS) regression methods. They used total hydrophobicity, molecular mass (log⁡*M*), and residual numbers to develop their regression models, and the amino acid *z*-scores, namely, *z*_1_: hydrophobicity, *z*_2_: bulkiness/molecular size, and *z*_3_: electronic property, were calculated using the method developed by Hellberg et al. [20] (individually or in combination with the mentioned parameters) to develop a PLS model (Table 1, number 30). They concluded that the developed models for whole data sets and data subsets (comprising different peptide length) were significant and improved for subsets [3]. Moreover, the combination of *z*-scores and studied variables improved the correlation of predicted and observed values for di- and tripeptides. 

QSBR model development for three or more amino acid peptides has not been studied as well as that of dipeptides. This work aims to build suitable models for predicting the bitterness of 224 peptides and 5 amino acids on the basis of their calculated structural parameters. Structural parameters (0D–3D) were calculated using Dragon [28] software. The selected parameters were used to develop linear and nonlinear models, and the prediction capability and robustness of the proposed models were studied using internal and external validation methods according to the QSAR method validations guidelines [29] and references [30–33]. The impacts of the selected parameters and structural features on bitterness were evaluated according to the parameter definitions. 

## 2. Materials and Methods

### 2.1. Data Set

A total of 229 experimental bitterness values (224 peptides and 5 amino acids) determined by human sensory evaluations were obtained from [3]. The details of peptide sequences and the bitterness activity expressed as a log⁡(1/*T*) (*T* being the bitter threshold concentration (*M*)) are summarized in Table 2. The ranges of bitterness were 1.0–5.7, and the numbers of amino acids of the studied peptides were 2–14.

### 2.2. Calculated Descriptors

The 2D structures of all molecules were drawn and converted to 3D structures using the HyperChem 7 software. The completed model and the molecular mechanics energy minimized molecules were used as inputs for the Dragon 5.4 software [28]. The software calculated 20 subsets of molecular descriptors, including 2D autocorrelation, 3D-MoRSE descriptors, centered fragments, Burden eigenvalues, connectivity indices, constitutional descriptors, edge adjacency indices, eigenvalue-based indices, functional group counts, geometrical descriptors, GETAWAY descriptors, information indices, molecular properties, Randic molecular profiles, RDF descriptors, topological charge indices, topological descriptors, walk and path counts, and WHIM descriptors. The structural parameters calculated after discarding the constant and near-constant values (1295 descriptors) were saved and further analyzed using the SPSS 11.5, STATISTICA 7 and MATLAB 7.8 software.

### 2.3. Outlier Detection

In order to identify possible outliers, two different methods of principal component analysis (PCA) mapping and standard scores were used. According to the nature of the outliers, which could be related to the different mechanism of binding because of the different structural features, recording errors, or inaccurate design of samples, no single outlier detection approach could identify all kinds of outliers [34]. In this study, two approaches, one in descriptor space (PCA plot of scores) and the second in response space (standard score), were used to check the outliers before the main numerical analysis [35].

### 2.4. Training-Test Selection

The training set plays an important role in developing the properties of the model, as the more similar the molecules for training the model are, the more accurate the expected results are. Thus, the selection of the training and test sets is one of the most important steps in model development and should be done so that each set reflects the original data set as much as possible. *K*-means clustering is one of the most frequently used methods of data set splitting. This procedure identifies relatively homogeneous groups of molecules (each observation has the nearest distance to the mean of the cluster) based on selected properties (biologic activities and structural parameters). Using this method, we divided the data set into 10 clusters, and three subsets (i.e., training, test, and validation sets) were selected from them. The validation set was excluded from the study before the descriptor selection step, and the test set was excluded before the model development step. 

### 2.5. Descriptor Selection

In order to reduce the dimension of the variable matrix, a correlation analysis was carried out, and both the highly correlated and constant variables were excluded. A home-developed MATLAB toolbox was employed that considered the following criteria for excluding a variable: [1] the intercorrelation with other descriptors higher than 0.99; [2] the correlation with activity lower than intercorrelated descriptors; and [3] the frequency of repeated values for a descriptor (lower than 10% of cases).

After this step, a genetic algorithm-partial least square (GA-PLS) algorithm [36] was used to select the most significant variables. GA-PLS (a combination of genetic algorithm and PLS regression) was also developed and utilized as a variable selection method in QSAR and QSPR studies by Leardi [36] and applied for QSAR studies [37, 38]. The MATLAB 7.8 software was used to run the GA-PLS method developed by Leardi. The variables were divided into subgroups (containing up to 200 variables), and each subgroup with the corresponding log⁡1/*T* values (*Y*) was introduced to the algorithm as input. The output (containing descriptors scoring based on the cross-validated percentage of explained variance) was produced after 100 runs. As the results of the GA-PLS were a bit different for each run, the top 50% scores of each subgroup were combined with the next 100 variables, and this step was repeated up to the final step. A comparative study was done in this study to check the capability of GA-PLS for descriptor selection in comparison with common methods (i.e., PLS and stepwise). Results showed that GA-PLS was able to extract more significant descriptors. The only drawback of this method was the scoring differences between different runs, which could be solved using the mentioned procedure (i.e., selection of 50% of each run instead of limited descriptors [37]).

**Figure 1:**
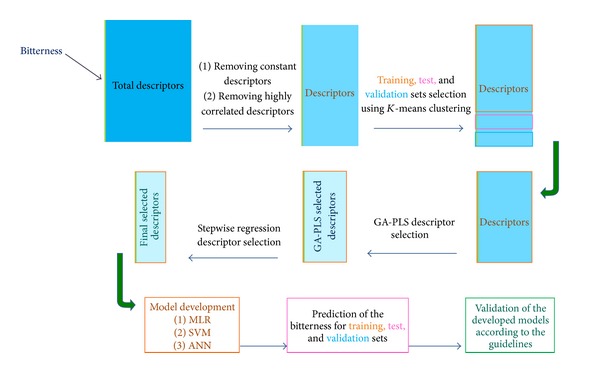
Procedure of descriptor selection, QSBR model development, and validation processes.

Twenty percent of high score variables of the final step were selected as the most significant variables and were further studied by stepwise regression and bivariate correlation analysis in which the selected variables using stepwise regression were investigated concerning their intercorrelations. The less intercorrelated variables were selected for the final model development process. The complete procedure of descriptor selection is summarized in Figure 1. 

### 2.6. Model Building

Linear and nonlinear models were developed using the selected descriptors. The details of the model development and validation process are discussed in the next sections.

### 2.7. Linear Model Using Multiple Linear Regression (MLR)

The selected parameters were used for developing QSBR equations using the multiple linear regression correlation (MLR) method, and the goodness of fit and statistical significance of the models were evaluated using *R* ^2^ (coefficient of determination), *F* (variance ratio), and the MPD (mean percentage deviation) values calculated using:

(1) $$\begin{array}{c} \text{MPD}=\frac{100}{N}\sum \left|\frac{Y_{\text{pred}.}-\;Y_{\text{exp.}(\text{mean})}}{Y_{\text{exp.}(\text{mean})}}\right|. \end{array}$$

The relative frequency of individual percentage deviations (IPD) was studied in order to define the model prediction capacity for each data point. The IPD was calculated using

(2) $$\begin{array}{c} \text{IPD}=100\left|\frac{Y_{\text{pred}.}-\;Y_{\text{exp.}(\text{mean})}}{Y_{\text{exp.}(\text{mean})}}\right|. \end{array}$$

In order to compare the MPD values with the relative standard deviations (RSD) between experimental bitter values measured by different research groups (inter laboratory relative standard deviations (ILRSD)), the ILRSD values for the available data were computed using

(3) $$\begin{array}{c} \text{ILRSD}=\frac{100}{N}\left|\frac{Y_{\text{exp.}}-\;Y_{\text{exp.}(\text{mean})}}{Y_{\text{exp.}(\text{mean})}}\right|. \end{array}$$

### 2.8. Nonlinear Models Using Support Vector Regression (SVR) and Artificial Neural Network (ANN)

In the next stage, the selected descriptors were used to derive non-linear models using SVR and ANN. To construct an ANN, the Levenberg-Marquardt algorithm [39] was used by nftool toolbox of MATLAB 7.8 software to train the network. Selected descriptors and the bitter activity of training sets were introduced as input and output values, respectively. The training set was randomly classified for training, validation, and test sets in order to avoid overfitting, and then networks were trained. SVR, another non-linear model, constructs a hyperplane in a multidimensional space that provides minimum error by employing a non-linear Kernel function. Some parameters such as capacity parameter (*C*); *ε* are related to type of noise in the data, and *γ* is related to radial base function (RBF), which is the most common type of Kernel functions. The SVM model and optimization of parameters were done using STATISTICA 7 software.

### 2.9. Model Validation

The developed models were evaluated using the leave-many-out (LMO) cross-validation method. The *q* ^2^ values were calculated using ((A.8)) of the appendix.

### 2.10. Chance Correlation

To check the possibility of a chance correlation, 10 times shuffled activities were correlated to the variables, and the produced regression coefficients were compared with the developed model regression coefficient.

### 2.11. External Validation of Proposed Models Using the External Test Set

A set of statistical criteria [30, 31] was employed to analyze 36 data points of the test set:

1. *R* ^2^ > 0.6,

where *R* ^2^ is the coefficient of determination between the predicted and observed values.

(2) (*R* ^2^ − *R*_0_^2^)/*R* ^2^ < 0.1 and 0.85 ≤ *K* ≤ 1.15 or (*R* ^2^ − *R*_0_^′2^)/*R* ^2^ < 0.1 and 0.85 ≤ *K*′ ≤ 1.15,

where *R*_0_^2^ is the coefficient of determination obtained using predicted values relative to a regression line fit for experimental values and required to pass through the origin, and *R*_0_^′2^ is the corresponding coefficient obtained using experimental values relative to a regression line fit for predicted values and required to pass through the origin. *K* and *K*′ are the slopes of regression lines through the origin for fits for experimental and predicted data, respectively.

(3) |*R*_0_^2^ − *R*_0_^′2^| < 0.3.

In addition, another criterion proposed by P. P. Roy and K. Roy [32] was considered as

(4) $R_{m}^{2}=R^{2}\left(1-\sqrt{R^{2}-R_{0}^{2}}\right)$,

in which *R*_*m*_^2^ > 0.5 indicates the good external predictability of the QSAR models.

## 3. Results and Discussion

### 3.1. Outlier Detection

According to standard score analysis, there is no outlier. The PCA map of scores showed that most of the data points fell into the acceptable data space, and there is no outlier in the studied data set.

### 3.2. Training-Test Selection

The selected training, test, and validation data sets are listed in Table 2 along with the computed accuracy criteria. The activity ranges for training, test, and validation sets were 1.00–5.40, 1.16–5.70, and 1.49–5.40, respectively. The number of data points for the training, test, and validation sets was 181, 36, and 10, respectively.

### 3.3. Descriptor Selection

A total of 1292 descriptors were calculated using Dragon 5.4 software. This number was reduced to 244 descriptors after correlation analysis was performed using a home-developed toolbox. The remaining descriptors were analyzed using the GA-PLS method, and the best scoring descriptors (descriptors included in Table 3) were selected for further analysis using stepwise regression and bivariate cross-correlation studies (the intercorrelation between the selected descriptors was less than 0.9). After excluding the nonsignificant descriptors, the remaining descriptors were checked to find the possible intercorrelation, and the final descriptors were selected using a stepwise regression method (see Table 4). The final six selected descriptors were again checked for intercorrelation. Six descriptors are suitable for the construction of a QSAR model [40].

### 3.4. Evaluation of the Selected Descriptors

**Figure 2:**
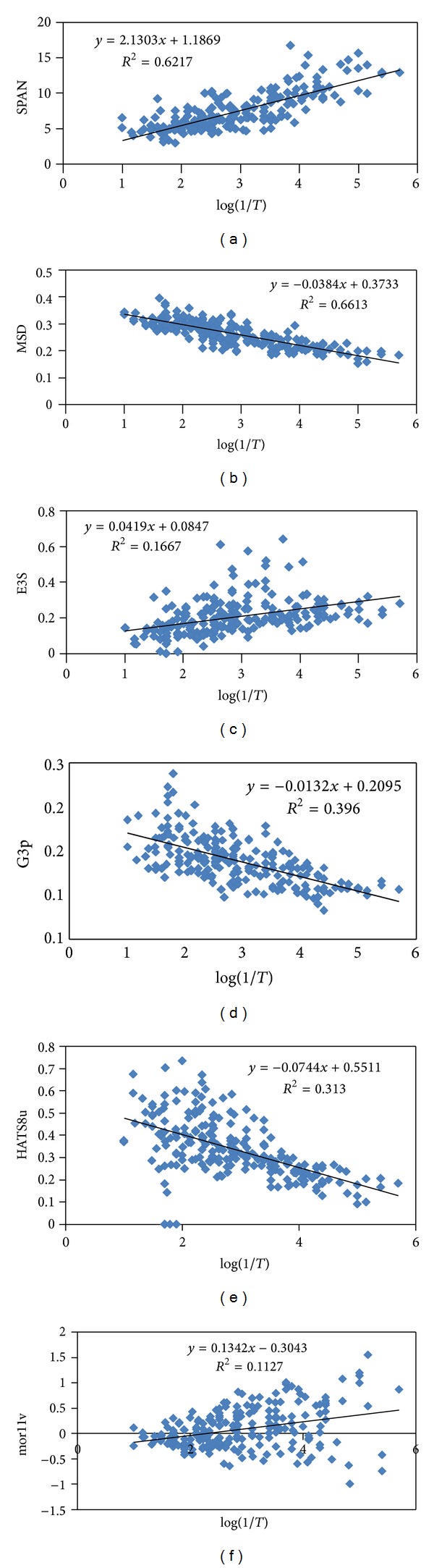
Correlation of selected descriptors with bitter activity (*R* ^2^ is coefficient of determination and is calculated using ((A.6)) of the appendix).

SPAN belongs to the size descriptors which evaluate the dimension of the molecule and often calculate it from the molecular geometry. This descriptor is a suitable size descriptor for macromolecules [41], and its selection for the studied data set with the developed method showed the method to be suitable for descriptor selection. The correlation study results (Figure 2) showed that bitterness increased upon the enhancement of the SPAN (Figure 2(a)). This finding is in agreement with previous findings [1–13] which suggested a significant correlation between peptide size and bitterness. The investigation of the correlation of peptide subsets with this descriptor showed less correlations in comparison with the whole dataset. Therefore, SPAN can be regarded as a general descriptor rather than a specific one for peptide subsets. Thus, SPAN can be used in the primary steps of peptide bioactive compound discovery to recognize bitter compound fractions.

Mean square distance (MSD) index (Balaban) [28, 41] contributes negatively to bitterness (Figure 2(b)). MSD decreases with the increasing of molecular branching in an isomeric set [41]. Furthermore, it decreases with an increase in the number of atoms. This is in agreement with the findings on the increase in bitterness following an increase in the number of amino acids in the studied peptides. The lower correlation coefficient between peptide subsets by MSD in comparison with the total dataset showed that MSD is a general bitterness indicator rather than a subset bitterness descriptor.

Bitterness increases with the enhancement of E3s (Figure 2(c)). E3s is one of the accessibility directional WHIM indices which is weighed by atomic electrotopological states [28, 41]. Indeed, this descriptor defines size, shape, polarizability, and the conformational properties of the studied molecules together. Its correlation with the bitterness is weaker than MSD and SPAN descriptors, but its removal from the model decreases the correlation coefficient significantly. It is not a strong identifier for utilization in drug development processes. 

G3p, the 3rd-component symmetry directional WHIM index (weighed by polarizability) [28, 41], showed a negative correlation with bitterness (Figure 2(d)). Along with E3s, these descriptors contribute to the electrical properties of the molecule. G3p decreases with the increase in the number of amino acids. In fact, more bitter compounds possess larger G3p values. Similar to the SPAN and MSD descriptors, the correlations with subsets are less than the general dataset, and this descriptor can be regarded as a general identifier. HATS8u (Figure 2(e)) belongs to the GETAWAY descriptors. These descriptors are molecular descriptors derived from the molecular influence matrix (MIM) [12, 28]. HATS, indices known as spatial autocorrelation based descriptors, encode information nonstructural fragments [28, 41]. They are suitable for describing differences in a congeneric series of molecules. The effective position of substituent and fragments in molecular space and information about the molecular size and shape can be encoded by unweighted HATS indices. Moreover, they are independent of molecule alignment. 

In summary, GETAWAY descriptors encoded local information while WHIM descriptors related to the holistic information of the molecules; thus, their joint uses are advised. HATS8u decreases with an increase in molecular size. The negative relation of this descriptor with bitterness is in agreement with the findings about the size and bitterness relation.

3D-MoRSE descriptors (3D molecule representation of structures based on electron diffraction) are derived from infrared spectra simulation using a generalized scattering function [28]. Mor11v, weighed by van der Waals volume, belongs to these descriptors which can be regarded as an indicator of size, mass, and volume of the molecules. By decreasing the size of the peptide, the Mor11v values tend toward zero (Figure 2(f)). In fact, the absolute values of Mor11v decrease by the decrease in size of the molecule. The absolute quantity of it worsens the model, and it should be used in the model in the present form including negative and positive values for different peptides. The only similar trend was observed for alogp values of the peptides. The negative Mor11v values belong to the molecules with negative alogp values (less hydrophobic peptides). 3D-MoRSE descriptors cannot encode the lipophilicity of the molecules. The overall correlation of bitterness to this descriptor is positive [28]. 

### 3.5. Model Building Using Linear Model (MLR)

**Figure 3:**
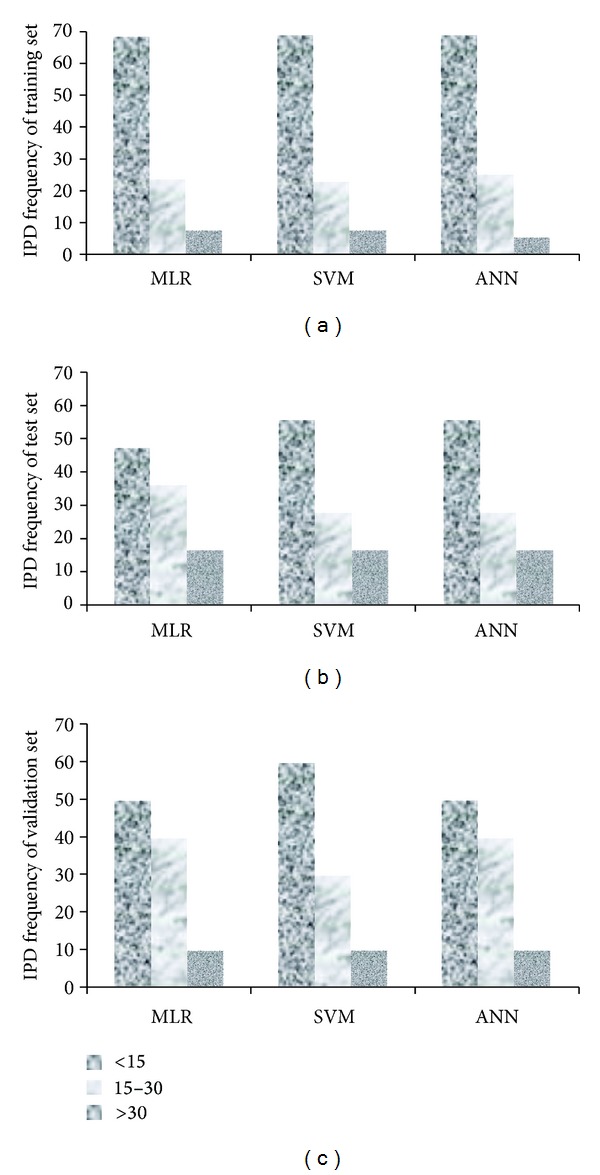
IPD frequencies (IPD < 15, IPD = 15–30, and IPD > 30) of the training (top), test (middle), and validation (bottom) sets for MLR, SVM, and ANN models.

The selected descriptors were used to develop six MLR equations containing 1–6 descriptors. The adjusted *R* ^2^ showed that all descriptors improved the model fitting, the *R* ^2^ values accurately depicted the fitting improvement, and the best-fitted model could be rewritten as follows:

(4) log⁡(1T)=5.45(±0.63)+0.10(±0.02)SPAN +0.32(±0.09)Mor11v −7.88(±1.25)MSD −1.55(±0.30)HATS8u −5.39(±2.1)G3p+0.92(±0.35)E3s,R2=0.81,  F=125.73,S.E.P.=0.08,  MPD=13.7(±13.2),ND=181,

where S.E.P. stands for standard error of prediction and ND shows the number of data points. The MPD for test and validation sets were 18.1 (±15.5) (*N* = 36) and 16.9 (±12.6) (*N* = 10), respectively. The relative frequency analysis of the prediction errors showed that more than 50% of data can be predicted by the prediction error of less than 15%, which is acceptable for biological measurements, where the mean ILRSD for bitter activities of 19 dipeptides were measured by different research groups and were 12.1 (±10.7)%. In addition, the IPD frequency trend (Figure 3) is similar for training and test sets. The MPD for the peptides by log⁡(1/*T*) values less than 2.0 was 24.3 (±22.2) and for the peptides with log⁡(1/*T*) more than 2.0, it was 11.1 (±7.9). The highest IPD value (100.0%) in the training set was calculated for GR (log⁡(1/*T*) = 1.00), and in the test set, it was calculated for PGR (IPD = 65.4% and log⁡(1/*T*) = 1.60). In other words, the developed model was not able to predict the bitterness of less bitter peptides, and the selected descriptors are specific for the evaluation of the bitter peptides' interactions with the receptor.

LOO cross-validation was done using the PLS toolbox of MATLAB software. The *q* ^2^ value and RMSE were 0.76 and 0.44, respectively. The *q* ^2^ values for LMO cross-validation are reported in Table 3. The ranges of *q* ^2^ values were 0.61–0.88, which was in an acceptable range compared with *R* ^2^ values. The ranges of corresponding *R* ^2^ and adjusted *R* ^2^ for the desired subsets were 0.80–0.82 and 0.79–0.82, respectively (see Table 5). Chance correlation (*Y* randomization) analysis was done using 10 times shuffled bitter activity, and the results (*R* ^2^ = 0.01–0.10) rejected the possibility of fortune correlation (see Table 6).

### 3.6. Model Building Using Nonlinear Models (ANN and SVM) and Comparison with the Linear Model (MLR)

**Figure 4:**
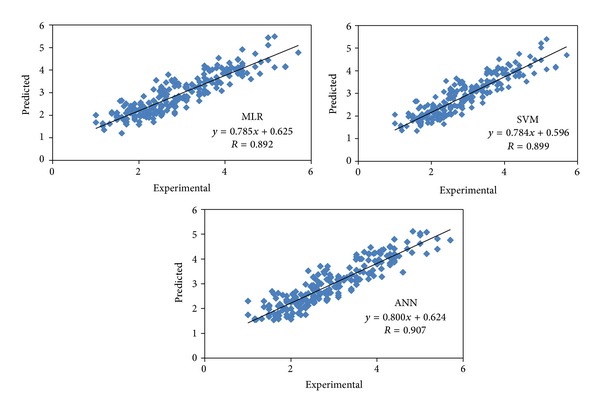
Experimental versus predicted plots for linear and nonlinear models. (*R* is calculated using ((A.7)) of the appendix).

The six selected descriptors were introduced to ANN as input values and the bitter activity as output data, and the networks were developed using the Levenberg-Marquardt algorithm [38]. The number of hidden layers was three. In addition, SVM models were developed using STATISTICA 7 software. Using the training set, three parameters of SVM were optimized by 10-fold cross-validation. The optimized values of *C*, *ε*, and *γ* were 91, 0.07, and 0.06, respectively. The MPD values of the proposed models for training, test, and validation sets were 13.8, 16.8, and 16.9 for MLR; 13.0, 16.0, and 15.0 for SVM; and 13.1, 16.1, and 14.8 for ANN, all of which are in acceptable ranges, and there is no significant difference between these subsets. The IPD frequencies (Figure 3) revealed that both SVM and ANN methods produced more accurate results, especially for the test sets. The plots of experimental versus predicted values (Figure 4) confirmed the discussed results.

### 3.7. External Validation of the Proposed Models

Statistical criteria of external test sets are depicted in Table 7. The results show that the proposed models using linear and nonlinear models passed the proposed statistical criteria in the literature, and they are robust and valid for external prediction.

### 3.8. Comparison with Previous Model

The only similar study was published by Kim and Li-Chan [3]. They used the amino acid three *z*-scores along with three parameters (total hydrophobicity, residue number, and log⁡ mass value) to develop a PLS model. A comparison of the newly developed model with their model is summarized in Table 8. It should be noted that the aim of this paper was to develop a general model rather than different models for different subsets, and reported correlation coefficients belong to the general model which was computed for subsets. The comparison of the corresponding *R* values showed that the developed model could represent the bitter activity variance of three and more peptides better than the PLS model, while for dipeptides the PLS model produced more accurate results. Developed SVM and ANN models resulted in more accurate results for the total data set compared with both the PLS and MLR models (Tables 1 and 5; Figure 4).

## 4. Conclusion

General MLR, SVM, and ANN models were developed to predict the bitterness of 229 peptides and amino acids. The capability of the MLR model to reveal the impact of each descriptor on bitter activity was its main advantage, where more accurate predictions by SVM and ANN made them suitable models for precise predictions. Obviously, individual models (i.e., models developed for each peptide subset) produced less prediction errors, but considering the convenience of application of the general models, such models are preferred during the primary stages of peptide production and evaluation. The developed models can be used in nutraceutical and pharmaceutical industries.

## Figures and Tables

**Table 1:** Details of previously developed QSBR models.

No.	Descriptors	Model	*q* ^2^ (CV)	*R* ^2^	RMSE	Ref.
1	MS-WHIM	PLS	0.633	0.704	nd*	[18]
2	ISA-ECI	PLS	Nd	0.847	nd	[19]
3	*z*-scales	PLS	0.713	0.824	0.26	[20]
4	GRID	PLS	0.780	nd	nd	[21]
5	MHDV	PCR	0.864	0.919	0.18	[22]
6	MEEV (M3)	MLR	0.588	0.773	0.34	[23]
7	MEEV (M4)	MLR	0.677	0.734	0.34	[23]
8	SSIA-AM1	PLS	0.837	0.85	0.25	[24]
9	SSIA-PM3	PLS	0.829	0.888	0.22	[24]
10	SSIA-HF	PLS	0.798	0.844	0.25	[24]
11	SSIA-DFT	PLS	0.741	0.856	0.24	[24]
12	MARCH-INSIDE	MLR	0.860	0.881	0.23	[25]
13	Constitutional	MLR	0.820	0.846	0.26	[25]
14	Topological	MLR	0.890	0.91	0.20	[25]
15	Molecular	MLR	0.580	0.618	0.39	[25]
16	BCUT	MLR	0.720	0.783	0.30	[25]
17	Galvez	MLR	0.560	0.617	0.40	[25]
18	2D	MLR	0.710	0.753	0.32	[25]
19	Randic	MLR	0.512	0.559	0.42	[25]
20	Geometrical	MLR	0.895	0.909	0.20	[25]
21	RDF	MLR	0.814	0.851	0.25	[25]
22	3D-MoRSE	MLR	0.880	0.914	0.20	[25]
23	GETAWAY	MLR	0.857	0.889	0.22	[25]
24	WHIM	MLR	0.799	0.861	0.25	[25]
25	SZOTT	PLS	0.736	0.908	0.20	[26]
26	VSW	PLS	0.696	0.868	0.24	[16]
27	V	MLR	0.921	0.948	0.17	[15]
28	E	MLR	0.888	0.940	0.21	[27]
29	E	SVR	0.912	0.962	0.12	[17]
30	*z*-scores	PLS	0.800	0.850	nd	[3]

*No data.

**Table 2:** Details of the observed-predicted (using MLR, SVM, and ANN methods) bitterness and corresponding errors (IPD).

Peptide sequence	Observed	MLR	IPD	SVM	IPD	ANN	IPD
Training data set							
R	1.60	1.20	25.1	1.81	13.4	1.90	18.5
F	1.70	1.86	9.3	1.83	7.5	1.86	9.5
P	1.90	1.87	1.6	2.36	24.3	2.16	13.5
L	1.70	1.82	6.9	1.82	7.3	1.86	9.4
V	1.70	1.62	5.0	1.61	5.3	1.68	1.1
GR	1.00	2.00	100.1	2.31	130.7	2.06	106.0
RR	2.11	2.38	12.9	2.69	27.4	2.84	34.7
PP	2.34	2.05	12.4	2.35	0.2	2.26	3.3
KP	2.52	2.39	5.0	3.28	30.1	3.20	26.8
PR	2.52	1.97	21.7	3.26	29.3	3.22	27.9

RF	2.60	2.68	3.0	2.81	8.1	2.76	6.0
RP	3.10	2.34	24.5	3.05	1.5	3.03	2.1
LI	2.40	2.07	13.8	2.12	11.5	2.01	16.4
KF	2.04	2.46	20.8	1.83	10.5	1.84	10.0
VF	2.52	2.46	2.5	2.32	8.0	2.36	6.3
VY	2.52	2.44	3.1	2.74	8.8	2.77	9.9
YG	2.52	2.37	6.1	2.48	1.7	2.39	5.3
YY	2.63	2.82	7.2	2.47	6.0	2.47	6.0

FI	2.83	2.62	7.6	2.32	18.0	2.06	27.2
IF	2.83	2.54	10.2	3.58	26.6	3.52	24.4
YF	3.10	2.70	12.9	3.31	6.8	3.29	6.2
VA	1.16	1.63	40.9	1.55	33.9	1.49	28.6
VG	1.19	1.34	12.9	1.56	30.8	1.36	14.3
PA	1.32	1.62	22.5	1.59	20.5	1.55	17.2
IE	1.37	2.13	55.5	2.30	68.2	2.12	54.7
IQ	1.49	1.91	28.3	1.97	32.5	1.93	29.7
IS	1.49	1.80	20.7	1.67	12.4	1.80	20.7
IT	1.49	2.11	41.3	2.08	39.6	2.03	36.2

SL	1.49	1.99	33.7	2.14	43.8	1.92	28.7
WE	1.56	2.63	68.6	2.70	73.0	2.65	69.6
IK	1.65	2.40	45.5	2.42	46.5	2.33	41.3
IA	1.68	1.77	5.5	1.64	2.3	1.66	1.1
AL	1.70	1.52	10.9	1.78	4.7	1.77	3.8
VV	1.71	1.59	6.9	1.70	0.5	1.63	4.8
LA	1.72	1.84	6.9	1.72	0.1	1.76	2.3
PY	1.80	2.09	15.9	1.93	7.3	2.02	12.1
GW	1.89	2.18	15.5	2.00	5.7	2.15	13.9
PL	2.22	1.98	10.9	2.21	0.2	1.95	12.0

PI	2.33	1.85	20.5	2.10	9.9	1.92	17.6
IP	2.40	2.06	14.1	2.25	6.3	2.03	15.3
YL	2.40	2.52	5.2	2.61	8.9	2.55	6.1
LY	2.46	2.79	13.4	2.89	17.3	2.76	12.2
IW	3.05	2.84	6.9	2.86	6.3	2.76	9.5
FY	3.13	2.80	10.4	2.90	7.5	2.84	9.2
LW	3.40	2.63	22.6	2.80	17.8	2.86	16.0
IV	1.90	1.80	5.3	2.31	21.5	2.06	8.2
FV	2.23	2.55	14.4	2.29	2.8	2.06	7.4
VE	2.23	1.99	10.6	2.57	15.4	2.46	10.3

YP	1.70	2.51	47.8	1.72	1.0	1.57	7.9
PK	2.23	2.06	7.8	2.27	1.7	2.10	5.9
LD	2.23	2.19	1.7	2.26	1.3	2.09	6.2
AD	2.23	2.15	3.7	2.22	0.5	2.00	10.5
LE	2.52	2.15	14.6	2.33	7.5	2.13	15.5
GE	2.83	2.13	24.8	2.63	7.2	2.58	8.8
VL	2.11	2.04	3.2	2.47	16.9	2.37	12.4
GP	1.79	2.28	27.3	2.09	16.7	1.94	8.6
FG	2.00	2.24	12.1	2.25	12.5	2.26	12.8
PF	2.14	2.28	6.4	2.34	9.3	2.29	6.9

GF	2.36	2.25	4.5	2.30	2.5	2.24	5.2
GY	2.15	2.14	0.4	2.12	1.5	2.17	0.8
LF	2.82	2.52	10.5	2.70	4.2	2.55	9.4
FL	2.85	2.58	9.4	2.64	7.4	2.57	9.7
GL	1.64	1.80	9.6	1.70	4.0	1.75	6.6
LG	1.71	1.70	0.7	1.58	7.7	1.70	0.6
IG	2.01	1.71	15.1	1.69	16.0	1.70	15.6
GI	2.17	1.91	12.1	1.82	16.3	1.84	15.4
LL	2.47	2.21	10.5	2.33	5.6	2.08	16.0
II	2.54	2.05	19.1	2.24	11.9	2.05	19.1

IL	2.54	2.12	16.6	2.29	9.9	2.08	18.2
RGP	1.90	2.51	31.8	1.66	12.4	1.83	3.9
FGG	2.34	2.27	3.2	2.10	10.3	2.09	10.9
GFG	2.52	2.35	6.7	2.25	10.9	2.10	16.5
PPP	2.70	2.39	11.6	2.62	3.1	2.42	10.3
GLL	2.83	2.37	16.4	2.33	17.6	2.07	26.7
RPG	3.10	2.71	12.7	2.46	20.5	2.37	23.5
LGG	1.00	1.68	67.6	1.75	75.0	1.67	67.1
GGL	2.00	1.99	0.4	2.00	0.2	1.92	4.1
LGL	2.30	2.38	3.3	2.43	5.8	2.34	1.7

LLG	2.30	2.35	2.1	2.39	4.0	2.31	0.3
PIP	2.85	2.37	16.7	3.71	30.1	3.51	23.2
LLL	2.92	2.70	7.5	3.06	4.8	2.94	0.9
GYG	1.70	1.72	1.3	2.63	54.5	2.42	42.2
GVV	2.34	2.05	12.4	2.98	27.3	3.07	31.3
VVV	2.34	2.38	1.8	1.76	24.9	1.64	29.8
PPF	2.63	2.48	5.7	3.38	28.4	3.38	28.5
YGG	2.63	2.60	1.3	2.67	1.6	2.46	6.3
RPF	2.83	3.28	15.9	2.55	9.8	2.44	13.8
FGF	2.92	2.85	2.4	3.24	11.1	3.29	12.5

DLL	3.10	3.18	2.6	2.63	15.2	2.67	13.7
GFF	3.23	3.04	5.9	3.14	2.8	3.08	4.7
FPF	3.40	3.31	2.8	3.38	0.6	3.25	4.4
GYY	3.40	3.02	11.2	3.15	7.4	3.06	9.9
PFP	3.40	2.88	15.3	2.74	19.5	2.58	24.2
YYY	3.70	3.45	6.8	3.73	0.7	3.57	3.5
GGV	1.48	1.93	30.2	1.75	18.4	1.79	21.0
PGG	2.34	1.69	27.6	2.02	13.6	2.00	14.6
GGF	2.83	2.42	14.4	2.39	15.4	2.36	16.8
GGP	2.04	1.95	4.4	2.60	27.3	2.51	23.1

PPG	2.04	2.22	8.6	2.35	15.4	2.16	5.7
FFG	2.65	2.93	10.5	3.06	15.5	2.99	12.8
ELL	3.40	2.91	14.5	2.73	19.7	2.82	17.0
FFF	3.70	3.44	7.1	4.28	15.7	3.94	6.4
EGG	2.83	2.06	27.2	2.71	4.2	2.61	7.9
YGY	3.10	2.84	8.5	2.87	7.4	2.79	10.0
YPF	3.52	3.03	13.9	3.18	9.6	3.04	13.7
GGLG	1.60	1.92	19.8	1.55	3.3	1.34	16.2
FGFG	3.52	2.94	16.4	3.55	1.0	3.56	1.2
GPFF	3.80	3.21	15.6	3.19	16.0	3.15	17.0

RPGF	3.80	3.39	10.9	3.41	10.2	3.36	11.6
FGGF	3.92	3.08	21.5	3.32	15.3	3.2	18.3
RPFF	4.40	3.82	13.2	4.42	0.4	4.24	3.6
GLGG	1.70	1.84	8.2	1.70	0.2	1.74	2.3
LGGG	1.90	1.98	4.1	2.26	18.9	1.92	0.8
PFPP	2.34	3.14	34.3	2.39	1.9	2.41	2.8
GPPF	2.52	2.88	14.2	2.56	1.7	2.47	1.9
RRPP	2.7	3.14	16.4	3.00	11.2	3.11	15.3
VYPF	3.52	3.46	1.6	2.97	15.7	3.05	13.5
PFIV	3.52	3.12	11.5	3.28	6.9	3.33	5.5

FFPR	4.00	3.87	3.2	3.79	5.2	3.87	3.3
FFPP	2.52	3.27	29.6	2.51	0.3	2.43	3.5
FFPE	2.76	3.56	28.8	3.49	26.3	3.55	28.6
GGFF	2.85	3.48	22.0	2.39	16.1	2.34	18.1
FFPG	2.90	3.38	16.6	3.37	16.3	3.35	15.6
LLLL	3.23	3.35	3.7	3.18	1.6	3.23	0.0
FFGG	2.52	3.20	27.0	2.41	4.4	2.31	8.2
RRPFF	4.70	4.15	11.7	4.69	0.3	4.63	1.4
GGGLG	1.90	2.28	19.9	2.34	23.1	2.08	9.4
GLGGG	1.90	2.15	12.9	1.77	7.0	1.69	10.8

LGGGG	1.90	2.16	13.6	2.51	32.3	2.49	31.1
GGVVV	2.11	2.84	34.6	2.21	4.7	2.02	4.5
FFPGG	2.83	3.58	26.6	3.18	12.4	3.18	12.2
PGPIP	3.11	3.25	4.6	3.26	4.8	3.19	2.6
RGPPF	2.63	3.45	31.2	2.95	12.2	2.89	9.8
PPFIV	2.92	3.30	12.9	2.64	9.6	2.69	7.9
RPGFF	3.51	3.82	8.8	3.98	13.4	3.86	9.9
RPGGFF	4.04	3.95	2.3	4.28	5.8	4.06	0.6
PFPGPI	3.36	3.63	8.1	3.65	8.7	3.56	6.0
RPFFGG	3.92	4.11	4.7	4.22	7.6	4.07	3.9

RGPPGF	3.52	3.31	5.9	3.55	0.9	3.43	2.6
RGPPFI	4.60	3.60	21.7	3.47	24.7	3.52	23.4
RGPFIV	4.30	3.84	10.7	3.83	10.9	3.76	12.6
RGGFIV	3.10	3.35	8.0	3.02	2.6	2.89	6.7
RGPPFF	4.23	4.04	4.6	4.13	2.4	3.98	5.9
RRPPGF	4.40	3.73	15.3	4.79	9.0	4.75	7.9
RRPPFF	5.15	4.13	19.9	5.08	1.4	5.40	4.9
GPPFIV	2.92	3.40	16.6	3.28	12.4	3.20	9.5
GGFFGG	3.70	4.02	8.8	3.72	0.4	3.55	4.0
KPPFIV	3.82	3.56	6.8	3.42	10.4	3.51	8.2

GGRPFF	4.04	4.07	0.6	3.98	1.5	3.83	5.1
PVLGPV	3.30	3.25	1.6	3.26	1.2	3.21	2.8
FPPFIV	3.52	3.66	4.0	4.18	18.8	4.09	16.3
RGPPGGV	2.48	3.07	23.9	2.93	18.2	2.96	19.2
RGPPGFF	4.40	3.97	9.7	4.04	8.3	3.93	10.7
RGPPFFF	5.00	4.45	11.1	4.62	7.6	4.47	10.6
VIIPFPG	3.60	4.08	13.3	3.49	3.1	3.5	2.8
VIFPPGR	4.10	4.33	5.5	3.87	5.6	3.83	6.6
RGPPGIG	2.78	3.49	25.5	3.41	22.7	3.43	23.5
RGPPGGF	3.08	3.25	5.4	3.17	3.0	3.10	0.8

YPFPGPI	3.80	4.09	7.7	4.21	10.7	4.05	6.6
RPPPFFF	4.70	4.73	0.6	4.41	6.2	4.22	10.2
VIPFPGR	4.15	4.13	0.5	4.38	5.6	4.23	1.9
PFPGPIP	3.60	3.54	1.6	4.00	11.2	3.98	10.7
RGPPGFG	3.68	3.56	3.1	3.47	5.7	3.49	5.2
RGPFPIV	3.95	3.85	2.5	3.92	0.7	3.88	1.9
RPFFRPFF	5.00	5.11	2.2	4.96	0.9	5.03	0.6
RGPKPIIV	4.08	3.87	5.0	4.21	3.2	3.83	6.3
VYPFPPGI	3.82	4.23	10.7	4.21	10.1	4.15	8.7
RGPEPIIV	4.51	3.87	14.2	4.12	8.7	3.80	15.8

RGPPGGFF	4.11	3.37	18.0	3.28	20.1	3.28	20.3
GGRPFFGG	4.40	4.30	2.3	3.92	10.8	3.81	13.3
RGPPGGGFF	3.95	4.05	2.6	4.24	7.4	4.09	3.5
GGRGPPFIV	4.10	3.95	3.6	4.35	6.1	4.30	4.9
RGPPFIVGG	4.31	4.06	5.7	4.04	6.3	4.01	7.1
FFRPFFRPFF	5.15	5.49	6.7	4.21	18.2	4.06	21.1
PVRGPFPIIV	5.40	4.16	22.9	4.82	10.8	4.18	22.6
VYPFPPGINH	4.30	4.48	4.3	4.51	4.9	4.38	2.0
VYPFPPGIGG	3.52	4.20	19.2	3.08	12.4	3.00	14.9
VYPFGGGINH	3.64	3.96	8.7	4.01	10.1	3.88	6.6

RPFFRPFFRPFF	5.00	5.44	8.9	5.04	0.8	5.22	4.4
RGPPFIVRGPPFIV	4.40	4.92	11.8	3.87	12.0	3.75	14.8
PVLGPVRGPFPIIV	4.83	4.36	9.7	5.11	5.9	4.48	7.2

Test data set							
RG	2.11	1.97	6.4	2.29	8.4	2.01	4.6
AV	1.16	1.71	47.6	1.59	37.4	1.58	36.3
ID	1.37	2.07	51.4	2.07	51.2	1.97	43.9
VD	1.90	1.94	2.2	2.36	24.5	2.15	13.4
LV	2.23	1.89	15.1	1.75	21.6	1.82	18.4
VI	2.23	1.90	14.7	1.99	10.9	1.96	12.1
FP	2.77	2.54	8.3	2.65	4.3	2.46	11.1

FF	3.01	2.75	8.6	2.89	4.0	2.82	6.5
AF	1.81	2.41	33.4	2.55	40.7	2.39	31.8
PGR	1.60	2.65	65.4	2.60	62.3	2.65	65.9
RRR	2.40	3.55	48.0	3.52	46.8	3.55	47.8
FIV	2.83	2.77	2.1	2.68	5.2	2.71	4.2
GGY	2.83	2.44	13.8	2.55	9.7	2.45	13.5
GRP	3.10	2.64	14.8	2.54	18.1	2.64	15.0
YYG	3.20	3.10	3.2	3.17	0.9	3.11	2.9
KPF	3.40	3.18	6.4	3.14	7.6	3.11	8.5
GLG	2.00	1.58	21.3	1.57	21.5	1.69	15.7

GPG	1.70	2.09	23.2	2.11	24.0	2.01	18.1
KPK	2.52	2.97	17.7	2.82	11.8	3.02	19.9
VYP	2.52	2.97	17.7	2.87	13.9	2.90	15.0
GGGL	2.34	2.26	3.2	2.39	2.1	2.10	10.0
RPFG	3.41	3.47	1.8	3.37	1.1	3.34	1.9
RGFF	3.80	3.69	3.0	3.95	4.0	3.83	0.7
GGLGG	1.90	2.29	20.6	1.93	1.7	1.92	0.9
GGGGL	2.65	2.21	16.6	2.40	9.5	2.17	17.9
PGPGPG	2.60	3.10	19.2	3.14	20.6	3.09	19.0
PFPIIV	3.90	3.43	12.0	3.55	9.0	3.40	12.7
VIFPPG	2.68	3.80	41.8	3.71	38.4	3.66	36.6

RGPPFIV	4.30	3.86	10.3	3.75	12.7	3.78	12.1
VYPFPPG	3.52	4.06	15.4	4.09	16.1	3.95	12.2
RGPFPIIV	5.40	4.14	23.3	4.40	18.5	4.14	23.4
RGPGPIIV	4.81	3.84	20.3	4.26	11.5	3.88	19.2
RRPPPFFF	5.70	4.78	16.2	4.76	16.5	4.69	17.6
VIIPFPGR	3.85	4.54	18.0	4.47	16.1	4.51	17.1
VYPFPPIGNH	4.30	4.46	3.7	4.48	4.1	4.38	1.8
GGRGPPFIVGG	4.40	4.21	4.3	4.21	4.4	4.11	6.6

Validation data set							
IN	1.49	2.11	41.9	2.12	42.4	2.01	34.8
WW	3.60	2.99	16.9	3.18	11.7	3.05	15.3

GV	1.74	2.22	27.8	2.19	25.8	2.08	19.8
FPK	2.52	3.10	22.9	2.94	16.7	3.14	24.7
FPP	2.34	2.65	13.3	2.56	9.6	2.62	12.1
PGP	2.04	2.56	25.4	2.57	26.0	2.41	18.0
VIF	2.89	2.78	3.8	2.79	3.6	2.82	2.3
RPPFIV	4.10	3.74	8.7	3.61	12.0	3.65	11.0
RGPPFGG	3.23	3.37	4.5	3.23	0.1	3.34	3.3
RGPPFIIV	4.30	4.12	4.1	4.20	2.4	3.99	7.2

**Table 3:** GA-PLS selected descriptors along with descriptive for training data set (181 peptides).

Name	Range	Minimum	Maximum	Mean	Std. deviation
rdf025p	112.70	4.07	116.77	30.90	21.13
mor15u	3.81	−1.90	1.91	0.18	0.65
eeig08x	4.21	−0.24	3.97	2.16	1.21
mor11m	2.13	−1.21	0.92	−0.15	0.40
rdf025v	108.21	4.00	112.20	29.52	20.28
mor15e	3.73	−1.57	2.16	0.26	0.70
mor21m	3.86	−3.93	−0.08	−0.90	0.64
belp7	1.88	0.00	1.88	1.18	0.42
rdf075m	144.42	0.00	144.42	16.28	24.18
mor31p	1.85	0.14	1.98	0.54	0.36
ggi6	3.87	0.00	3.87	0.77	0.74
mor11v	2.54	−1.00	1.55	0.10	0.40
mor31v	1.59	0.11	1.70	0.45	0.31
ncs	29.00	1.00	30.00	7.26	5.50
ti1	6217.22	−87.25	6129.97	255.45	699.86
e3s	0.64	0.00	0.64	0.21	0.10
gmti	1318857.00	347.00	1319204.00	81064.04	173637.51
vep1	8.18	2.61	10.78	5.28	1.63
ats1p	2.88	1.81	4.68	3.12	0.60
smtiv	775601.00	341.00	775942.00	48407.38	103187.64
behv6	2.84	0.96	3.79	2.82	0.53
alogp	8.07	−2.81	5.26	0.38	1.45
rtp_a	25.63	4.90	30.53	13.02	5.31
c002	18.00	0.00	18.00	4.13	3.38
j3d	10.70	2.41	13.10	5.35	2.46
idmt	1426115.69	182.31	1426298.00	75860.86	184400.59
mats2m	0.32	−0.16	0.16	0.04	0.06
mor11p	2.80	−1.29	1.51	0.05	0.43
mats2v	1426115.69	182.31	1426298.00	75860.86	184400.59
hats8u	0.70	0.00	0.70	0.33	0.13
smti	477598.00	168.00	477766.00	29053.36	63311.78
mor05u	36.43	−38.23	−1.80	−10.14	6.51
hats8e	0.68	0.00	0.68	0.34	0.13
mats2e	0.31	−0.15	0.15	0.03	0.06
l1u	53.45	2.41	55.86	12.98	9.33
rbn	46.00	1.00	47.00	11.61	8.03
h6m	1.81	0.00	1.81	0.34	0.36
rdf080e	387.99	0.00	387.99	39.41	61.82
rtv_a	23.23	3.99	27.21	11.50	4.82

**Table 4:** Intercorrelation of final descriptors.

	log(1/*T*)	SPAN	Mor11v	MSD	HATS8u	G3p	E3s
log(1/*T*)	1.00						
SPAN	0.79	1.00					
Mor11v	0.34	0.09	1.00				
MSD	−0.81	−0.78	−0.20	1.00			
HATS8u	−0.56	−0.52	−0.25	0.38	1.00		
G3p	−0.63	−0.64	−0.13	0.64	0.30	1.00	
E3s	0.41	0.22	0.31	−0.38	−0.16	−0.25	1.00

**Table 5:** Leave-many-out cross-validation results for MLR model.

Subset	*R* ^2^	*R*_adj_^2^	*q* ^2^
1	0.80	0.79	0.88
2	0.80	0.79	0.87
3	0.80	0.79	0.86
4	0.80	0.80	0.82
5	0.80	0.80	0.82
6	0.81	0.80	0.75
7	0.81	0.80	0.78
8	0.82	0.81	0.76
9	0.82	0.81	0.84
10	0.82	0.82	0.61

**Table 6:** Chance correlation results.

Shuffled *Y*	*R* ^2^	Shuffled *Y*	*R* ^2^
*Y*1	0.01	*Y*6	0.02
*Y*2	0.07	*Y*7	−0.02
*Y*3	0.01	*Y*8	0.03
*Y*4	−0.02	*Y*9	0.01
*Y*5	0.01	*Y*10	0.00

**Table 7:** Statistical parameters for test sets of MLR, SVM, and ANN models.

Statistical criteria	MLR	SVM	ANN
*R* ^2^ > 0.6	0.723	0.739	0.767
(*R* ^2^ − *R*_0_^2^)/*R* ^2^ < 0.1	0.001	0.002	0.004
0.85 ≤ *K* ≤ 1.15	0.999	1.010	0.989
|*R*_0_^2^ − *R*_0_^′2^| < 0.3	0.109	0.113	0.106
*R*_*m*_^2^ > 0.5	0.704	0.712	0.722

**Table 8:** Developed MLR model statistics for subsets of peptides compared with a previously developed model.

Data set	Developed model					Previous model	
	ND	*R* ^2^	*R*_adj_^2^	*R*	RMSE	*R* (PLS)	*R* ^2^ (PLS)
Dipeptides^a^	76	0.51	0.47	0.72	0.41	0.63	0.40
**D** **i** **p** **e** **p** **t** **i** **d** **e** **s** ^b^	**45**	**0.62**	**0.56**	**0.79**	**0.44**	**0.91**	**0.83**
**D** **i** **p** **e** **p** **t** **i** **d** **e** **s** ^c^	**47**	**0.59**	**0.53**	**0.77**	**0.42**	**0.85**	**0.72**
Three peptides	51	0.65	0.60	0.81	0.38	0.71	0.50
Tetrapeptides	23	0.72	0.62	0.85	0.48	0.90	0.81
Pentapeptides	12	0.89	0.80	0.94	0.37	0.88	0.77
Hexapeptides	20	0.65	0.48	0.80	0.47	0.75	0.56
Heptapeptides	16	0.79	0.68	0.89	0.38	0.95	0.90
Octa-tetradecapeptides	24	0.57	0.42	0.76	0.44	—	—
Whole data set^d^	227	0.80	0.79	0.89	0.46	0.81	0.66
Test and validation sets	46	0.76	0.73	0.87	0.56	—	—
Training set	181	0.81	0.81	0.90	0.43	—	—

^a^Average of experimental values was used when there were different values in different references.

^b^Experimental data were taken from different references.

^c^Experimental data were taken from [3].

^d^The *R* values for whole data set using SVM and ANN methods are 0.90 and 0.91, respectively.

## Appendix

(A.1) $$\begin{array}{c} \text{RMSE}=\sqrt{\frac{{\sum \left(Y_{\text{pred}.}-Y_{\text{exp.}(\text{mean})}\right)}^{2}}{N},} \end{array}$$

where *N* denotes the number of data points and *Y*_pred._ and *Y*_exp._ are the predicted and observed log⁡1/*T* (*T* is the bitter threshold concentration (*M*)) values

(A.2) $$\begin{array}{c} {\text{MSS}}_{\text{Reg}.}=\frac{\sum {\text{SS}}_{\text{Reg}.}}{\text{df}}, \end{array}$$

(A.3) $$\begin{array}{c} {\text{MSS}}_{\text{Res}.}=\frac{\sum {\text{SS}}_{\text{Res}.}}{\text{df}}, \end{array}$$

where SS is sum of squares, MSS is mean square, Reg. is regression, Res. is residual and cv is cross validated

(A.4) $$\begin{array}{c} {\text{MSS}}_{\text{Total}}={\text{MSS}}_{\text{Reg}.}+{\text{MSS}}_{\text{Res}}, \end{array}$$

(A.5) $$\begin{array}{c} F=\frac{{\text{MSS}}_{\text{Reg}.}}{{\text{MSS}}_{\text{Res}.}}, \end{array}$$

(A.6) $$\begin{array}{c} R^{2}=1-\frac{{\text{MSS}}_{\text{Res}.}}{{\text{MSS}}_{\text{Total}}}, \end{array}$$

(A.7) $$\begin{array}{c} R={\left(1-\frac{{\text{MSS}}_{\text{Res}.}}{{\text{MSS}}_{\text{Total}}}\right)}^{0.5}, \end{array}$$

(A.8) $$\begin{array}{c} q^{2}=R_{\text{cv}}^{2}=1-\frac{{\text{MSS}}_{\text{Res}.\text{cv}}}{{\text{MSS}}_{\text{Total}}}. \end{array}$$
